# Analysis of the structure of scientific knowledge on wine tourism: A bibliometric analysis

**DOI:** 10.1016/j.heliyon.2023.e13363

**Published:** 2023-02-01

**Authors:** Bartolomé Marco-Lajara, Javier Martínez-Falcó, Luis A. Millan-Tudela, Eduardo Sánchez-García

**Affiliations:** Management Department, University of Alicante, Spain

**Keywords:** Wine tourism, Bibliometric analysis, Wine industry, Knowledge structure

## Abstract

This research offers a bibliometric analysis of 588 publications on wine tourism published between 1998 and 2021, highlighting the years of publication, the publication format, the fields of research, the authors, the institutions, the main journals and the country of origin of both the authors and the scientific production analyzed. To our knowledge, no bibliometric study has focused on the study of wine tourism, so this study aims to fill this research gap, serving as a reference guide for both academics and professionals in the wine sector who want to know in depth how the study of this type of tourism has been approached. The results of the research show that the academic study of wine tourism dates back to the end of the 1990s, with the New World countries standing out in its study, especially Australia as the country with the highest scientific production and the largest number of academics focused on the study of this type of tourism. The study allows us to highlight the value of academic articles as the main means of disseminating research results, these being focused on tourism, business, economic and cross-cutting research. The results reveal relevant conclusions for academics, winemakers and tourism managers on the knowledge structure of wine tourism activity.

## 1 Introduction

Wine tourism represents the appropriate marriage of wine production and activity, being broadly defined as that experience associated with visiting vineyards, wineries and wine demonstrations in which wine tasting is the main element [1]. Thus, while wine production is based on winemaking, wine tourism focuses on attracting visitors, thus acting as a support channel for direct sales in wineries.

Today, wine tourism is one of the most important and most promising types of tourism, given that it is linked to the new consumption patterns of tourists, based on the importance of the experience, as well as on a shorter duration and greater frequency in the number of visits [2]. In Australia, which is the world's fifth largest wine-producing region, more than eight million tourists visit wineries each year, with more than one million wine tourists travelling to Australia from overseas, a figure that has tripled in the last two decades [3]. In the case of California (United States of America), the Napa Valley alone generated $54.8 billion for the state in 2021, as well as 875,000 direct and indirect jobs [4]. These countries are included in the so-called New World countries and are the pioneers in terms of vineyard technification and innovative practices in the development of wine tourism [5]. Old World wine-producing countries, on the other hand, such as France, Italy and Spain, are characterized by the maintenance of traditional methods of wine production, as well as a low level of dynamism in wine tourism [6]. However, wine tourism is increasingly seen as a strategic element in the wine-producing countries of the Old World [7].

The growing importance of wine tourism, given its recognized capacity to increase winery sales and favor rural development, has aroused the interest of academics from different disciplines, such as geography, economics, sociology and business management. The first research in the field of wine tourism dates back to the nineties of the last century, focusing on the influence of wine tourism activity in rural areas and on the behavior of wine tourists [8]. Thus, among the pioneering works on wine tourism, the books *Wine Tourism Around the World* [1] and *Explore Wine Tourism* [9] stand out for their relevance and disruptive character in the field, as well as the research carried out by Charters & Ali-Knight [10], Carlsen [11], Getz & Brown [12] and Mitchell & Hall [13].

From the early 1990s to the present day, a great deal of research has been published on the subject of wine tourism, mostly carried out by universities in collaboration with tourism organizations and the wine industry, in order to meet the needs of wineries [14]. Therefore, given the amount of scientific production generated around the field of wine tourism, it is necessary to analyze and classify the knowledge developed in this field in order to detect the main research fronts. More specifically, a series of indicators are used to estimate both the distribution and the intensity of work in the area by different agents (authors, institutions, publishers and countries), as well as the existing relationships between them. At the same time, the aim is to carry out a brief analysis of the temporal evolution of research on the subject of interest in order to know both the first existing references and their progression from there on. The efforts made in this research are aimed at establishing a reference point on which to base the search for bibliography on the subject under study, as well as to understand the structure of the scientific knowledge generated around wine tourism.

This research contributes to the literature in several ways. Firstly, to the best of our knowledge, despite the existence of narrative and systematic reviews on wine tourism activity, there is no bibliometric study regarding this topic, which represents an opportunity to further advance knowledge on wine tourism. Secondly, the bibliometric analysis has been carried out since the first academic publication on the subject in the end of the 90s of the last century until 2021, thus being a comprehensive analysis of the structure of knowledge on wine tourism from the first indexed research in the field until the present day. Thirdly, the research can serve as a reference guide for both academics and professionals in the wine industry who want to know in depth how the study of this typology of tourism has been approached, thus being a reference study for both researchers who are starting out in the study of wine tourism and for those experienced in the subject.

After this brief introduction, Section 2 shows the research gap to be covered. Section 3 presents the set of steps to achieve the objectives set out, Section 4 shows the results and, finally, Section 5 presents the main conclusions, limitations and future lines of research.

## 2 Literature reviews in the field of wine tourism

As the scientific production in the field of wine tourism has increased, it has become necessary to collect documents and clarify the research fronts in the research field under study. Table 1 shows the publications in journals indexed in the main collection of the Web of Science database (WoS) that aim to review the literature on wine tourism, classifying the reviews by their authors, the journal in which they have been published, the title of the article, the type of review, the number of papers analyzed, the period covered by the review and the countries to which the scientific production belongs.

**Table 1:** Reviews indexed in the main collection of the Web of Science on wine tourism. Source: own elaboration.

Authors	Journal	Title	Type of review	Papers analyzed	Period analyzed	Countries analyzed
Carlsen [11]	Journal of Wine Research	A review of global wine tourism research	Narrative review	59	1998–2004	Old World and New World
Charters [6]	Tourism: An International Interdisciplinary Journal	New World and Mediterranean wine tourism: A comparative analysis	Narrative review	36	1990–2009	Old World (Mediterrean countries) and New World
Mitchell & Hall [13]	Tourism Review International	Wine tourism research: the state of play	Systematic review	201	1992–2006	New World (Australia, New Zealand, Canada and United States)
López-Guzmán et al. [17]	Cuadernos de Turismo	Review of the scientific literature on wine tourism in Spain	Narrative review	58	1992–2012	Old World (Spain)
Durán-Sánchez et al. [21]	Estudios y Perspectivas en Turismo	Wine Tourism: Scientific Literature Analysis in Cross-cultural Research of Doctoral Thesis	Systematic review	57	1998–2015	Old World (Spain, Portugal, United Kingdom and France)
Montella [14]	Sustainability	Wine tourism and sustainability: A review	Systematic review	43	1992–2015	Old World and New World
Ramos et al. [18]	Worldwide Hospitality and Tourism Themes	Main challenges, trends and opportunities for wine tourism in Portugal	Narrative review	16	2000–2015	Old World (Portugal)
Anđelić et al. [22]	Economics of Agriculture	A review of wine and wine tourism presence in the scientific papers in journals in the field of tourism	Systematic review	91	1992–2019	Old World and New World
Gómez et al. [7]	Current Issues in Tourism	Wine tourism research: a systematic review of 20 vintages from 1995 to 2014	Systematic review	176	1995–2014	Old World and New World
Santos et al. [23]	Worldwide Hospitality and Tourism Themes	Wine and wine tourism experience: a theoretical and conceptual review	Narrative review	55	1992–2018	Old World and New World
Santos et al. [19]	PASOS: Revista De Turismo Y Patrimonio Cultural	Progress and prospects for research of Wine Tourism in Portugal	Systematic review	36	2003–2018	Old World (Portugal)
Nave et al. [20]	International Journal of Wine Business Research	A systematic literature review on sustainability in the wine tourism industry: insights and perspectives	Systematic review	60	2005–2020	Old World and New World

As can be seen, there are only 12 papers that have reviewed the literature on the subject of wine tourism, which highlights the need to continue analyzing the state of the art of the discipline. In this scientific production, more than half (58.33%) were published in the last five years (2017–2021 period), which shows that literature reviews on wine tourism have intensified recently. Likewise, of the 12 reviews, 5 are narrative and 7 are systematic in nature. It is worth highlighting the high number of narrative reviews (41.67%) on the subject despite the limitations of this type of review, given that, among other aspects, in these reviews the author's subjective criteria prevails when selecting the works, the data found in the different publications are not quantitatively synthesized and the procedure followed to obtain the information is not specified [15]. The rest of the reviews follow a qualitative systematic approach, given that they present the scientific production in a descriptive way without any advanced statistical analysis (systematic review without meta-analysis). This type of review represents an advance with respect to narrative reviews, given that their reproducibility is guaranteed, as the steps followed to obtain the scientific output under analysis are explicitly and clearly stated [16].

With regard to the period of analysis of the reviews carried out, it is important to highlight that all the reviews begin their analysis from the late 1980s and early 1990s, a period considered as the beginning of the academic study of wine tourism, up to the year in which the reviews were carried out. Likewise, most of the reviews analyze less than a hundred articles (83.33%), with the exception of the reviews by Mitchel & Hall [13] and Gómez et al. [7], in which 201 and 176 articles are analyzed respectively. Furthermore, academic literature focused on wine tourism in both Old and New World countries predominates. However, while Mitchell & Hall [13] focus only on the scientific production of New World countries (Australia, New Zealand, Canada and United States), López-Guzmán et al. [17], Ramos et al. [18], Santos et al. [19] focus on Old World countries, such as Spain and Portugal. In addition, although the main objective of the reviews is to analyze the scientific production related to wine tourism, Montella [14] and Nave et al. [20] specifically study the scientific production linking wine tourism activity and sustainability.

The analysis of the literature reviews on wine tourism has served to identify three main shortcomings in this type of research. Firstly, there is a small number of literature reviews on the activity of wine tourism, since there are only 12 articles that have addressed this objective. Secondly, there is no bibliometric analysis of the discipline, as the existing reviews are narrative and exploratory systematic reviews. Thirdly, the last year analyzed in the reviews carried out was 2020, so that the analysis can be updated to the current time. These shortcomings justify the need to develop our study, given that this research aims to contribute to the generation of new knowledge in the field of wine tourism, carrying out a bibliometric analysis from the end of the 1990s until 2021. The work therefore makes it possible to overcome the existing gap in knowledge, as it contributes to the generation of new knowledge through a bibliometric analysis that comprises from the origins of the discipline of wine tourism to the present day.

## 3 Methodology

### 3.1 Searching procedure

In order to analyze the knowledge structure regarding wine tourism, a systemic and replicable procedure must be developed and explained. For this reason, PRIMSMA guidelines were considered on the design procedure of the bibliometric review carried out. This declaration was designed to help the authors document in a transparent manner the rationale for the review, as well as the process followed to reach the research results [24]. Based on its guidelines, the following actions have been carried out: (1) the source of information used is the Web of Science database (WoS). In the case of systematic and bibliometric reviews, two main strategies can be followed: the first one consists of the use of a single source of information (prioritizing the homogeneity on results), while the second one allows the capture of more results as different databases are used, but at the expense of heterogeneity and the need for controlling potential duplicities. The authors of this study have chosen the first one. (2) Regarding the database used, WoS has been the chosen one by its recognition by researchers. More concretely, the Core Collection from WoS has been used due to the following reasons given by its owner and developer company [25].

• Around 21,100 journals indexed
• 250 disciplines covered
• Access to proceedings and books
• Broad information included on each indexed record
• Unification of institution names

With the use of the WoS Core Collection, the authors pretend to make this analysis replicable over time, thus allowing other researchers both to update the results exposed above in this work and detect changes over time in an easy way, so those that have never developed a bibliometric analysis can do so. If several databases were used, different specific software should be used, thus hindering replicability for those with no previous experience.

(3) Once the source of information had been chosen, the next step was to determine the search equation that returns the best results. This is a reiterative process as a broad equation is tested and, through simplification and comparison with previous equations, that which gives proper results is defined as the definitive one. As part of the process is creative and based on trial an error, details around this action transcends the aim of the paper, but the procedure can be requested to the authors.

After the previously mentioned process, the following equation has been chosen as the final one:

TS= ((cellar NEAR/0 door$ OR wine*) NEAR/5 (event$ OR trip$ OR touris* OR leisure OR holiday$ OR vacation$))

As can be seen, the equation is divided in two main parts. The first one states the entity of study while the second one allows to precise the activity developed in the corresponding entity. In order to give coherence to the results, the search operator NEAR/5 has been used, restricting valid records to those that present (at least) a concept from every part separated by no more than five words [26]. Regarding each of the parts of the equation, the operator OR has been applied in them not to restrict results to just one of the concepts included, as well as wildcards [25] to capture all possible variations of the concepts stated in the equation. It must also be stated that the search equation is applied to the topic (TS), which in WoS consists of the work title, abstract and keywords (both work and the ones added by WoS), so the results returned by the database have more guarantees of being topic-related.

(4) In a second phase, another restriction has been applied. In order to avoid potential records that have no relation with wine tourism (e.g. medical research regarding the effect of wine consuming), they must belong to, at least, one of the following WoS categories^1^: Hospitality Leisure Sport Tourism, Management, Business, Environmental Studies or Economics. By doing so, two things are achieved: the abovementioned relation with the study topic and the possibility to discover collaborations among these areas with the rest that have not been explicitly exposed.

The stated process, once applied, returned a total of 588 results that date from 1998 to 2021.

Finally, it should be noted that no review protocol was developed. However, as can be seen in the present and previous sections, the research clearly describes the reason for the review, the objectives and methods used to locate the scientific production, as well as the collection and analysis of the data from the included studies. Thus, by specifying the reason why the protocol document was not prepared, the requirements 24a and 25 b of the PRISMA regulations related to the registration and protocol of the results are fulfilled.

### 3.2 Variables considered

Once the search equation has been finally chosen, variables to be considered were stated. As the main goals of this work are exposing the knowledge structure about wine tourism as well as its research development over time, features that describe production distribution and time evolution are needed. In the following sections, the variables used, as well as the reasons and data treatment and modification regarding them, are exposed.

#### 3.2.1 Year of publication

The first of the variables considered is the time each of the records returned by the search equation were published (in journals, proceedings, books and other formats). By doing so, the first works in the field can be detected, as well as changing trends in the interest of wine tourism research by variations in the amount of production. Regarding this variable, no changes nor revisions needed to be done as results given by WoS contained no major errors.

#### 3.2.2 Publishing formats

This variable is used to analyze how knowledge around wine tourism is spread from the research community to different stakeholders (including members from the community itself). In this sense, the means (formats) used are stated and compared in absolute and relative terms. It must be stated that a record, it is a work, can belong simultaneously to more than one publishing format, so the number resulting from the sum of each format is higher than the numbers of records returned by the equation. This situation is repeated in other variables and will be noted in their corresponding section.

#### 3.2.3 Research fields

Regarding research fields, there is a fact that must be exposed: as the searching procedure states that results must belong (at least) to the areas of Hospitality Leisure Sport Tourism, Management, Business, Environmental Studies or Economics, those will be the research fields with the most records. However, the interest of this section lies in analyzing the rest of research fields that collaborate with the ones previously exposed. In this case, the same as in publishing formats happens: a work can be linked to multiple knowledge areas. As it also happened in the previous variables exposed, no changes needed to be addressed.

#### 3.2.4 Authors

Exposing the authors involved in the topic studied in this work allows readers to know those researchers that bring considerable contributions to it. As can be deducted, authors are one of the main pillars that underpin knowledge production. Thus, deepening in the matter of who the most influential researchers in wine tourism are, not only the contribution from each of them is analyzed, but also the interaction among them through their research production. In order to do so, different approaches have been developed. The first one is the definition of the main authors, which are considered the most influential researchers in the area.

To define an author as a main one, the number of works have been first reviewed. The trend noted shows a group differentiated in this regard, it is, each of the authors in this group mainly show a different number of records. In comparison, those in a second group show little contributions (in quantity terms), sharing many of them the same number of records, what has been considered as an indicator for sporadic incursions in the topic field.

Once these groups have been defined, those authors in the first one have been manually checked to avoid the inclusion of authors that have not been real drivers of wine tourism research. This can mainly happen for two reasons. The first one is due to the authorship algorithm used by WoS. When a work is not manually linked to an author, an automatic computing program assigns that work to a researcher. This algorithm sometimes makes an incorrect assignment, or even creates an author that does not exist (the latter can be detected through the revision of each of the documents assigned to each supposed author). The second one takes place when an author has several records that belong to the same work (e.g. several book chapters in the same book). Consequently, both situations have been checked and corrected, withdrawing a total of 4 authors, two per each reason exposed above.

Once this has been done, researchers have been sorted by their number of records (contributions), followed by the number of citations (excluding their own) and, consequently, by the relative importance of their contributions (measured through a ratio comparing the number citations per work).

The above process has been developed in order to expose the individual contribution of each author. However, similarities in research production are also required to be approached. To do so, a co-citation study has been done with the use of the software VOSviewer in its 1.6.18 version. By doing so, authors that are cited together by other researchers are highlighted and, thus, similarities in their production are displayed through a network map.

#### 3.2.5 Institutions

As well as in the previous point, institutions or organizations returned by the search equation sometimes appear duplicated. It is particularly seen in the case of some Universities, where registers are indexed to the institution itself and one of their campuses at the same time. In the same way, a particularity appears in the case of the United States of America. USA universities are usually grouped by the called “state systems”, so the different universities from a state form a cluster that, in the case of WoS, generates duplicities as a record is linked to a university and its system at the same time.

In order to solve those problems, the first 50 organizations (the ones considered as the most influential by number of works related with wine tourism) have been deeply analyzed, checking if they have duplicities with the rest in the available results (which consist of 500 organizations). If any duplicities were detected, a manual correction was done. In this sense, the campus or system was withdrawn from the list, and their records were assigned to the remaining institution in case that they were not already linked.

Once the data was depurated, organizations have been ordered by the number of works in the study field, as well as a brief analysis regarding the countries they belong to.

#### 3.2.6 Journals

Related to journals, prior checks only consisted in the withdrawal of results that differ from journals and conference proceedings.^2^ The reason to do so is the following fact: as the latter are periodically developed, other means of publication considered by WoS (e.g. books) do not, and may alter the analysis. Consequently, the analysis by sources of publication has been previously developed, so a deeper study (explained in the following paragraph) can be done now.

As a list can be created sorting journals by number of works (records), a more interesting view is the one that analyses the interest in wine tourism for each editorial line. It is, the study of journals is focused on the main areas that are addressed by the top journals and, thus, a more consistent knowledge is developed through grouping results by those main areas. By doing so, more interesting conclusions may arise as the analysis is not just limited to a brief list of journals, but also the main lines of research from them are considered.

#### 3.2.7 Countries

The final variable studied is work distribution by territories. In this sense, the contribution force exerted by each country is first measured by the number of works, as well as pointing out the countries that have main authors. In a second phase, a co-authorship network analysis has been developed and studied so collaborations among different countries are exposed. By doing so, it is possible to track those interactions that create synergies and, thus, must be controlled by those interested in wine tourism research, as those collaborations can be considered nucleus of production.

Despite WoS offers quite depurated results, some of them are split (for example, United Kingdom is divided into England, Wales, Scotland, and Northern Ireland). Those kinds of situations have been manually solved, checking for duplicates and reassigning results if necessary.

### 3.3 Flow diagram about the systematic procedure

**Fig. 1:**
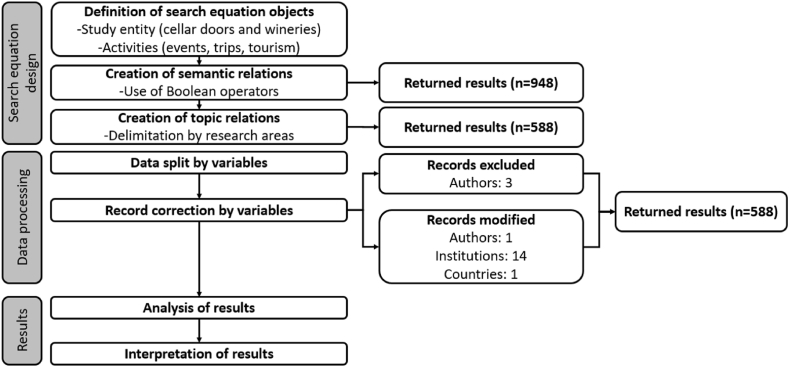
Flow diagram about the bibliometric review procedure developed. Source: own elaboration based on Preferred Reporting Items for Systematic Reviews and Meta-Analyses (PRISMA) Flow Diagram.

In order compile the previously stated actions, as well as the logic followed in the results section, a flow diagram chart comprising all the searching, sorting and following analyzing procedures is represented in Fig. 1.

## 4 Results

### 4.1 Year of publication

**Fig. 2:**
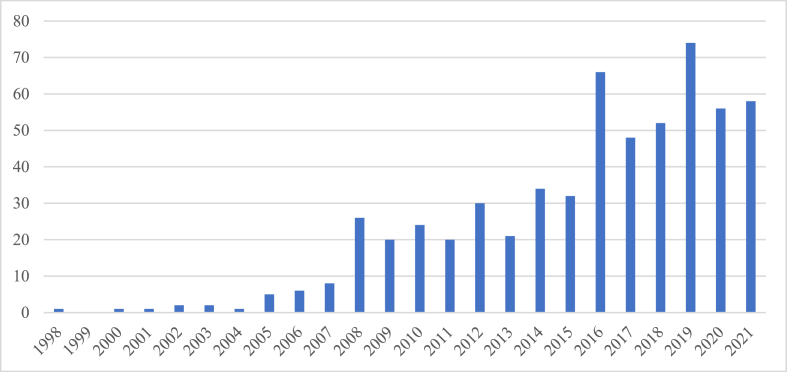
Wine tourism research production by year of publication. Source: Web of Science.

As Fig. 2 shows, the first indexed record dates from 1998, and corresponds to the journal article called *Relationship between organizational change and failure in the wine industry: An event history analysis* developed by Stoeberl et al. [27], where they study and state that organizational change does not affect business failure. Despite showing no record in 1999, from the year 2000 on, wine tourism research production has been developed uninterruptedly, having a notorious increase since 2008. Among the pioneering works in the field of wine tourism, as mentioned previously, are the books *Wine Tourism Around the World* [1] and *Explore Wine Tourism* [9], which are considered two cult books in the discipline, as well as the disruptive studies by Charters & Ali-Knight [10], Carlsen [11], Getz & Brown [12] and Mitchell & Hall [13].

Early research was mostly conducted by oceanic authors and focused on the link between wine tourism and the development of the rural areas in which the activity takes place. However, over time, new lines of research on the subject have been developed, with seven active research fronts in which the scientific production on the topic can be framed. These are: (1) territorial development, (2) wine routes, (3) the behavior of wine tourists, (4) the wine tasting experience, (5) wine festivals and festivities, (6) sustainability, (7) wine marketing in wineries.

### 4.2 Publishing formats

**Fig. 3:**
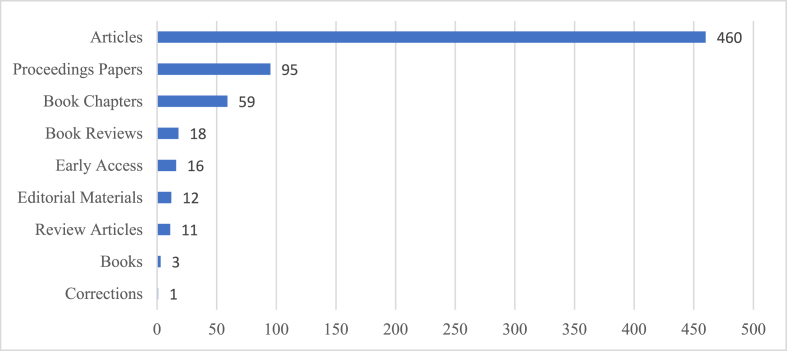
Number of records by publishing format used. Source: Web of Science.

Fig. 3 shows the production distribution by publishing formats used. As can be seen, journal articles are the main source for spreading wine tourism research with 460 results (that accounts for 78.2% of the 588 results returned by the search equation). Proceedings papers and book chapters are also noteworthy as the stand for 95 (16.2%) and 59 (10.0%) of the records respectively, showing that publishing format structure does not differ from other research areas. The rest of the results are divided among reviews, editorial materials, early access contents, books and even a work correction.

### 4.3 Research fields

Table 2 shows the work distribution by research fields. As can be expected, the first four fields correspond to the ones used to filtering in the search equation. However, the following ones show collaborations (and thus potential synergies) with the first ones. In this sense, economics in general turns out to be the most recurring area in this second group (59 results) as well as green sustainable science technology (52). It must also be highlighted the interest in environmental sciences (41) and regional urban planning (12) as some research lines regarding wine tourism not yet developed in these areas may emerge in the future (for example, the possible contribution on wine tourism to dynamization in depopulated areas), as many of the results seen in this fields just focus on sustainability.

**Table 2:** Number of results by research fields. Source: Web of Science.

	Field	Records		Field	Records
1	Hospitality Leisure Sport Tourism	409	16	Public Environmental Occupational Health	4
2	Management	108	17	Social Sciences Interdisciplinary	4
3	Business	99	18	Agriculture Multidisciplinary	3
4	Environmental Studies	72	19	Development Studies	2
5	Economics	59	20	Urban Studies	2
6	Green Sustainable Science Technology	52	21	Asian Studies	1
7	Environmental Sciences	41	22	Computer Science Information Systems	1
8	Agricultural Economics Policy	18	23	Computer Science Interdisciplinary Applications	1
9	Regional Urban Planning	12	24	Education Educational Research	1
10	Business Finance	10	25	History	1
11	Food Science Technology	9	26	Marine Freshwater Biology	1
12	Sociology	6	27	Oceanography	1
13	Geography	5	28	Operations Research Management Science	1
14	Area Studies	4	29	Public Administration	1
15	Computer Science Artificial Intelligence	4	30	Social Sciences Mathematical Methods	1

The results related to the research fields demonstrate the multidisciplinary nature of the field of study, since it has been analyzed how wine tourism can represent a new type of tourism to economically diversify a given territory (this topic being linked to Hospitality and Development Studies), to favor the preservation of the environment and the heritage in which the activity is carried out (being this topic linked to Environmental Studies and Environmental Sciences), to maintain and create employment in the wine-growing areas (this topic being linked to Economics Business Finance) or, among other aspects, to catalyze the processes of organizational innovation (being this topic linked to Management, Business and Green Sustainable Science Technology).

Despite existing more research fields that share works with the previous ones, the number of them are not high enough to confirm a current, strong collaboration. Nevertheless, some of those research fields show high potential to expand knowledge of the research topic addressed in this work. For example, those areas that are dedicated to the use and developing of new technologies may contribute by analyzing ways to optimize wine cellars by last state-of-the-art technologies to offer an immersive experience to clients. Development and urban studies can also be interesting for future research. As stated above, wine tourism can serve as a way to dynamize depopulated areas, as well as a diversification vector for those regions where the economy is mainly based in agriculture and wine producing, thus giving a chance to adopt activities from the service sector.

### 4.4 Authors

Once applied the procedure exposed in the methodology section, the obtained results are shown in Table 3. As can be seen, the main author is Abel Duarte Alonso with 30 records, being journal articles all of them, followed by Johan Bruwer (19 records) and Steve Charters (10). Regarding the country of the institution they belong to, it must be highlighted that 7 researchers are affiliated to Australian organizations, 5 to the Portuguese ones and 4 to New Zealand and the United States of America.

**Table 3:** Number of records, citations, work impact and affiliation country for each main author. Source: adapted from Web of Science.

	Author	Records	Citations^a^	Ratio	Country
1	Abel Duarte Alonso	30	304	10.1	Vietnam
2	Johan Bruwer	19	613	32.3	Australia
3	Steve Charters	10	516	51.6	France
4	Colin Michael Hall	8	187	23.4	New Zealand
5	Ian Phau	7	104	14.9	Australia
6	Tomás López Guzmán	7	95	13.6	Spain
7	Alessandro Bressan	7	89	12.7	Australia
8	Paulo Ramos	7	40	5.7	Portugal
9	Elisabeth Kastenholz	7	5	0.7	Portugal
10	Joanna Fountain	6	134	22.3	New Zealand
11	Robin M. Back	6	48	8.0	United States
12	Giuseppe Festa	6	35	5.8	Italy
13	Seng Kok	6	8	1.3	Australia
14	Donald Getz	5	555	111.0	Canada
15	Richard Mitchell	5	312	62.4	New Zealand
16	Jingxue Yuan	5	198	39.6	United States
17	Vlad Krajsic	5	84	16.8	Australia
18	Michelle O'Shea	5	84	16.8	Australia
19	Songshan Huang	5	49	9.8	Australia
20	Vasco Santos	5	25	5.0	Portugal
21	Seamus O'Brien	5	14	2.8	United Kingdom
22	Mar Gómez-Rico	4	142	35.5	Spain
23	Arturo Molina-Collado	4	142	35.5	Spain
24	Mark A. Bonn	4	112	28.0	United States
25	Evangelos Christou	4	63	15.8	Greece
26	Athina Nella	4	63	15.8	Greece
27	David A. Cohen	4	56	14.0	New Zealand
28	Qiushi Gu	4	47	11.8	China
29	Dan McCole	4	39	9.8	United States
30	Nuno Almeida	4	25	6.3	Portugal
31	Francesc Xavier Medina	4	24	6.0	Spain
32	Veronique Joukes	4	16	4.0	Portugal
33	Lino Meraz Ruiz	4	7	1.8	Mexico

a This column does not include own-citations.

**Fig. 4:**
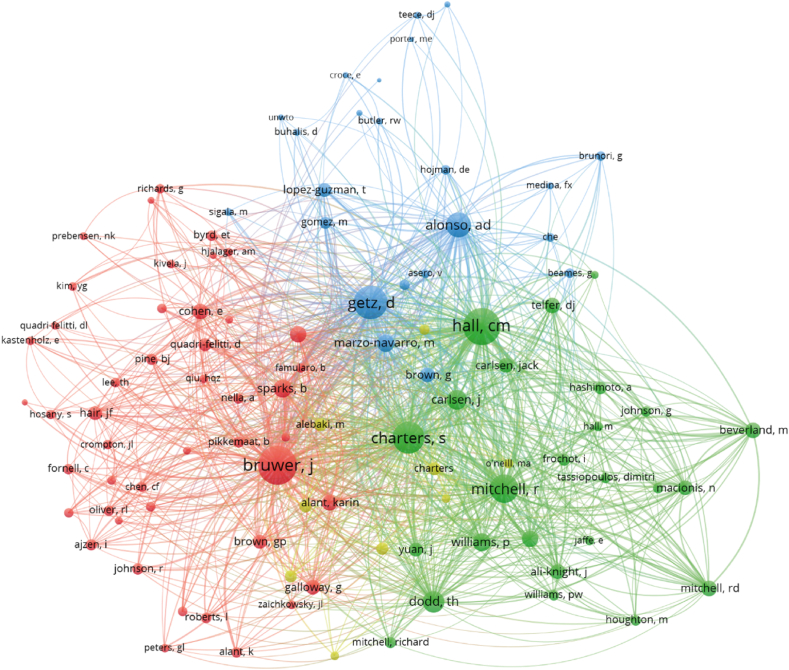
Co-citation network map for authors with a minimum of 25 citations. Source: Web of Science, edited with VOSviewer.

Despite being necessary to expose the content included in Table 3, the interest of author analysis remains in the co-citation relations shown in Fig. 4. The co-citation analysis developed allows to discover the number of times where two authors are cited together by other academics and, consequently, is used as a proxy for establishing potential relations in their research lines.

According to the number of co-citations, Abel Duarte Alonso and Johan Bruwer appear as the most influential in wine tourism research as they hold the highest number of co-citations with the rest of the main authors, thus following the same pattern shown in Table 3. Regarding the first author, his research in wine tourism focuses in both New-World (Australia and New Zealand) and Old-World (Spain) countries, sharing a variety of topics regarding wine tourism, as the works developed with authors such as Bressan, Kok and O'Brien in innovation [28] and sustainability [29,30], or those regarding wine tourism development with Bressan, O'Shea and Krajsic [[31], [32], [33], [34]]. Moving to Johan Bruwer, his most recurred line of research focuses on wine tourists' behavior such as dynamics, perceptions and experiences [[35], [36], [37], [38], [39], [40], [41], [42], [43], [44], [45]], authors that, despite some of them do not appear in Fig. 4, have collaborated with Bruwer, showing his influence in the area as he is related with a huge variety of researchers. As can be seen, the range of topics regarding wine tourism developed by these authors gives to their works the possibility to serve as a reference for many researchers in the area, allowing them to become the reference in the field.

### 4.5 Institutions

Once the pertinent corrections stated in the methodology section were developed, the results shown in Table 4 were obtained. As can be seen, the first three institutions (that are placed in Australia) show a notorious difference in terms of works compared to the rest of organizations. In fact, 6 of the top 10 institutions are established in Australian land, being Edith Cowan University the most outstanding in this sense. Other institutions that must be highlighted are those belonging to Old-World countries. For example, Universidade de Aveiro is recognized as one of the most important universities in wine research, something that explains its position in the above list. Remaining in Portugal, Universidade de Trás-os-Montes e Alto Douro has to be recognized as it has brought 10 works, most of them in the 2017–2022 period. Universidad de Córdoba (Spain) has considerable contributions through the research made by Tomás López Guzmán, being another research reference in the field. USA institutions also stand out, namely University of Central Florida (9 works), University of North Carolina (8) and Sonoma State University (6), with the latter being located close to the Napa Valley, the most recognized wine-producing area in the United States. Despite many institutions have been exposed in Table 4, a more interesting topic is the contribution of each country. In this sense, country ranking by number of top institutions is addressed in the subsection corresponding to regions.

**Table 4:** Institutions by number of records and region. Source: adapted from Web of Science.

	Institutions	Records	Region^a^
1	Edith Cowan University	31	Australia
2	Curtin University	24	Australia
3	University of South Australia	20	Australia
4	Universidade de Aveiro	12	Portugal
5	University of Adelaide	12	Australia
6	Universidad de Córdoba	11	Spain
7	Royal Melbourne Institute of Technology	10	Australia
8	Universidade de Trás-os-Montes e Alto Douro	10	Portugal
9	Western Sydney University	10	Australia
10	Hong Kong Polytechnic University	9	Hong Kong
11	Texas Tech University	9	United States
12	University of Canterbury	9	New Zealand
13	University of Central Florida	9	United States
14	University of North Carolina	8	United States
15	Griffith University	7	Australia
16	Lincoln University (New Zealand)	7	New Zealand
17	Universidade Fernando Pessoa	7	Portugal
18	University of Salerno	7	Italy
19	University of Turin	7	Italy
20	Instituto Universitario de Lisboa	6	Portugal
21	La Trobe University	6	Australia
22	Sonoma State University	6	United States
23	Universidade de Caxias do Sul	6	Brazil
24	University of Liverpool	6	United Kingdom
25	University of Otago	6	New Zealand
26	University of Queensland	6	Australia
27	Auburn University	5	United States
28	Instituto Politecnico do Cavado e do Ave	5	Portugal
29	Isla Santarem	5	Portugal
30	Liverpool John Moores University	5	United Kingdom
31	Michigan State University	5	United States
32	Neoma Business School	5	France
33	Universidad Autónoma de Baja California	5	Mexico
34	Universidad Autónoma de Chile	5	Chile
35	Universidad de Castilla-La Mancha	5	Spain
36	Universidad de Málaga	5	Spain
37	Universidade de Vigo	5	Spain
38	University of Aegean	5	Greece
39	University of Zaragoza	5	Spain
40	Autonomous University of Barcelona	4	Spain
41	Cornell University	4	United States
42	Florida State University	4	United States
43	Institute for Tourism (Croatia)	4	Croatia
44	Instituto Universitario da Maia - ISMAI	4	Portugal
45	Kedge Business School	4	France
46	Pennsylvania State University	4	United States
47	Polytechnic Institute of Viana do Castelo	4	Portugal
48	Southeast University (China)	4	China
49	Stellenbosch University	4	South Africa
50	Universidad de Cádiz	4	Spain

a The term *region* is used to include both countries and different legal terminologies as the case of Hong Kong, which is considered a *Special Administrative Region* of People's Republic of China.

The analysis of the institutions shows the importance of the New World institutions in the study of wine tourism, given that 7 of the top 10 institutions belong to this group of countries. This is due to the fact that it was the oceanic academics who first began to analyze the field of study given the relevance of wine tourism activity in those countries. However, the wine-producing countries of the Old World began to resemble more and more to those of the New World, since domestic wine consumption was increasingly lower, there was a lower propensity to import wine and wine was losing its value as a beverage associated with the diet of the European countries, to enhance the value of social stratification, vision and enjoyment associated with this beverage, as was the case in the countries of the New World. In this context, wine tourism activity became increasingly important for European countries, since it allowed them to face the new challenges faced by the sector, such as the lower consumption of domestic wine or its new perception. This importance aroused interest among academics in the Old World, leading to the generation of scientific production on the subject. Therefore, although the New World institutions are the first to have studies on the role of wine tourism in the territorial development of the rural regions in which wine tourism activities are carried out, at present, institutions belonging to both groups of countries develop research on the seven research fronts in the discipline mentioned in previous sections.

Finally, it must be also exposed that all institutions but one are universities. The exception is the Institute for Tourism in Croatia, which has contributed with 4 works in the area of wine tourism.

### 4.6 Journals

Once the withdrawal of non-valid records has been accomplished, the definitive list of journals is presented in Table 5. As can be seen, the journal Sustainability is the one with the highest number of contributions, followed by Pasos and Tourism Analysis. From the first 30 valid results, 28 are journals (in the sense of periodical publications), appearing only two conference meetings, one related with economy in general (journal number 26) and the other with tourism (number 27). 10 of the journals stated have a Journal Impact Factor (JIF) index, a measure applied to those journals that are considered to have enough quality and research impact [26]. Concretely, 6 belong to the 1st quartile in, at least, one of their categories, while 4 belong to the 2nd, meaning that they are placed in the 25% and 50% top journals among those that have previously been considered to have enough quality and impact.

**Table 5:** List of journals by number of records and their 2021 Journal Impact Factor (JIF) quartile. Source: adapted from Web of Science.

	Journal	Records	Highest 2021 JIF Quartile
1	Sustainability	41	Q2
2	Pasos: Revista De Turismo Y Patrimonio Cultural	25	n/d
3	Tourism Analysis	20	n/d
4	Tourism Management	16	Q1
5	Cuadernos de Turismo	15	n/d
6	Current Issues in Tourism	15	Q2
7	International Journal of Contemporary Hospitality Management	13	Q1
8	International Journal of Tourism Research	12	Q2
9	Tourism Review International	12	n/d
10	Tourism	11	n/d
11	Journal of Travel & Tourism Marketing	10	Q1
12	Tourism Recreation Research	10	n/d
13	Rosa dos Ventos: Turismo e Hospitalidade	9	n/d
14	Anatolia: International Journal of Tourism and Hospitality Research	8	n/d
15	Journal of Wine Economics	8	n/d
16	Tourism Planning & Development	8	n/d
17	International Journal of Culture Tourism and Hospitality Research	7	n/d
18	Journal of Destination Marketing & Management	7	Q1
19	Almatourism	6	n/d
20	Journal of Hospitality & Tourism Research	6	Q2
21	Tourism and Hospitality Research	6	n/d
22	Tourism Management Perspectives	6	Q1
23	Tourism Review	6	Q1
24	Investigaciones Turísticas	5	n/d
25	Journal of Business Research	5	Q1
26	Medunarodni Znanstveni Simpozij Gospodarstvo Istocne Hrvatske Jucer Danas Sutra	5	–
27	Proceedings of the 2nd International Conference on Tourism Research (2019)	5	–
28	Worldwide Hospitality and Tourism Themes	5	n/d
29	Zeitschrift fur Tourismuswissenschaft	5	n/d
30	Acta Turística	4	n/d

Moving on to their scope, it must be highlighted that most of the journals analyzed focus on tourism research. Indeed, and despite the specific of the topic field studied, only the Journal of Wine Economics is directly related with the economic sector studied, being the rest dedicated to business (e.g. Journal of Business Research), economic (e.g. Medunarodni Znanstveni Simpozij Gospodarstvo Istocne Hrvatske Jucer Danas Sutra) and cross-disciplinary works (e.g. Sustainability). This situation creates a gap to derivate wine tourism research to specialized journals and conferences in wine, as it may improve knowledge transmission to stakeholders in the sector. While tourism journals offer a wide range of topic options (that is useful to place wine tourism research in the radar), an increase of works in wine-related journals would allow to facilitate addressing to those in the sector.

### 4.7 Countries

**Fig. 5:**
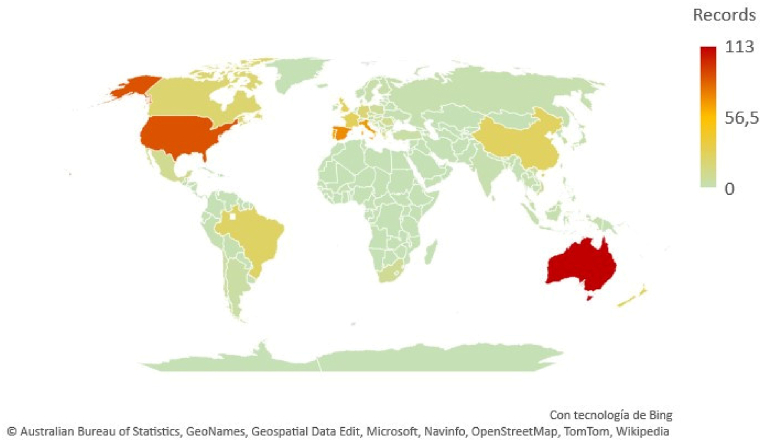
Number of records by countries. Source: Web of Science, edited with VOSviewer.

**Fig. 6:**
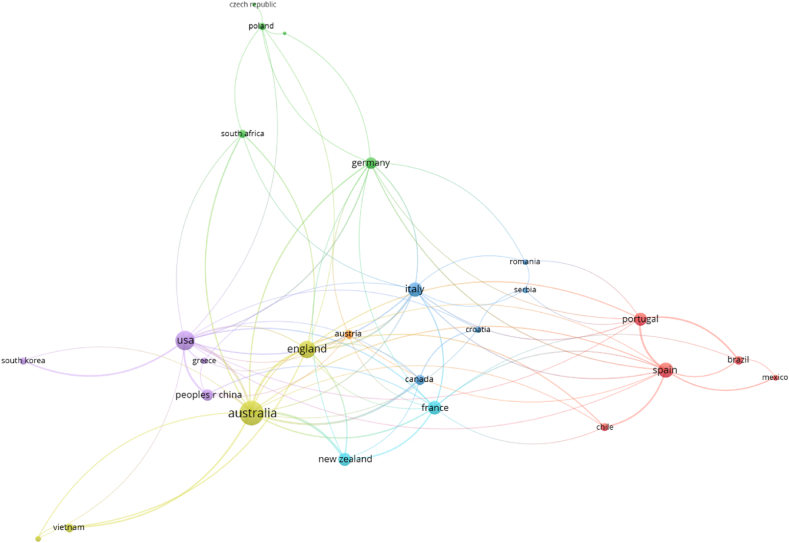
Co-authorship strength by countries (the force of collaboration is proportional to dot sizes) Source: elaborated from Web of Science data with VOSviewer software.

As can be seen in Fig. 5, Fig. 6, as well as in Table 6, Australia is the country with the highest contribution in terms of works and prominent authors. A total of 113 records (almost 1 in 5 works) are related to, at least, one Australian university, being the next country in this sense the United States, that accounts for the 15% of the production. Spain, Italy and France are also remarkable with more than 60 works each, having the rest of the countries less than half of that number. However, when moving to the number of main authors, New Zealand appears as the 3rd force (tied with Spain and the United States), what is also consistent with their number of institutions as 3 of the 8 universities are classified as top institutions in wine tourism research by the standard followed in this work (see section 4.5). Despite the contribution the rest of countries do in wine research (for example, South Africa is recognized as being a reference in this sense), their contribution in wine tourism research is diluted in comparison to the ones exposed, at least according to the search equation.^3^

**Table 6:** Countries by number of main authors affiliated to their institutions. Source: elaborated from Web of Science.

	Country	Main authors
1	Australia	7
2	Portugal	5
3	New Zealand	4
4	Spain	4
5	United States	4
6	Greece	2
7	Canada	1
8	China	1
9	France	1
10	Italy	1
11	Mexico	1
12	United Kingdom	1
13	Vietnam	1

Moving to mutual collaborations among countries (Fig. 6), Australia is again the one with the highest interaction with another territories (as the size of its point in the figure shows). In this sense, some of its collaborators (USA, United Kingdom and Spain) are also positioned as one of the most collaborating countries. In a second level, we find other European countries (Germany, Italy, France and Portugal), as well as New Zealand, China and Canada. In fact, collaborations of the last three mentioned are limited to 5 or less countries, showing the cutout line for this analysis.

With all the information abovementioned, it can be said that Australia is, up to date, the distinguished reference country in terms of wine tourism research, also being the USA, Spain, Italy, France and New Zealand considerable drivers in this research area.

## 5 Discussion and conclusions

The results presented in this research are of particular interest to the academic community, as well as to wine companies and professionals, since they contribute to understanding the structure of knowledge surrounding the study of wine tourism.

Firstly, the research shows that the academic study of wine tourism dates back to the late 1990s. This is in line with previous academic literature, which states that it was at the end of the last century when New World authors began to generate scientific production around this type of tourism [7]. In fact, despite the activity has been studied in both New-World and Old-World countries, the New World continues to predominate in the study of wine tourism, given that, of the five authors with the highest scientific production on wine tourism, four belong to that block of countries. Moreover, the three institutions with the highest scientific production are Australian (Edith Cowan University, Curtin University and the University of South Australia), which is also the country with the highest contribution in terms of outstanding works and authors. It can therefore be said that Australia is, up to date, the country of reference in terms of wine tourism research.

In fact, the importance of Australia as a country of reference in the study of wine tourism dates back to the beginnings of the discipline, since the first research on the subject was carried out mostly by Oceanic authors, focusing initially on the link between wine tourism and the development of the rural areas in which the activity takes place. However, over time, new lines of research on the subject have been developed, with seven active research fronts in which the scientific production on the topic can be framed. These are: (1) territorial development, (2) wine routes, (3) the behavior of wine tourists, (4) the wine tasting experience, (5) wine festivals and festivities, (6) sustainability, (7) wine marketing in wineries.

Secondly, the study shows that, despite the prominent role of the Oceanic country mentioned above, the United States, Spain, Italy and France are considerable drivers in this area of research, since, while the United States accounts for 15% of scientific production, Spain, Italy and France stand out with more than 60 papers each, the other countries having less than half that figure. In terms of cross-country collaborations, Australia once again stands out as the country with the most academic interaction with other wine-producing countries. However, the United States, the United Kingdom and Spain are also positioned as countries with a high level of academic collaboration for the study of wine tourism activity. From the results it can be concluded that there are two fundamental criteria by which academics collaborate with each other: (a) geographical proximity and (b) language affinity. On the one hand, the Australia-United States-England-New Zealand link stands out, as well as the Spain-Mexico-Chile link, which can be explained by the use of the same language (English and Spanish respectively). On the other hand, the Germany-Poland-Austria and Italy-Spain-Portugal block of collaborations may be explained by the geographical and cultural proximity between the regions.

Thirdly, scientific articles are the main source of dissemination in the field of wine tourism research, with articles in proceedings and book chapters playing a secondary role, which shows that the structure of dissemination of scientific knowledge in this field of research does not differ from other areas. The research results provide a list of the main journals for publishing research on wine tourism, which may be useful for researchers who are in the process of identifying scientific journals to disseminate their research results on wine tourism. As far as the scope of the journals is concerned, it is important to highlight that most of them focus on tourism research, the rest being focused on the publication of business, economic and cross-cutting works (detecting only a journal dedicated to wine research). This is an evidence of the need for wine journals to stimulate the dissemination of results on wine tourism activity.

The present research contributes new knowledge to the existing academic literature on the subject, given that, to the best of our knowledge, despite the existence of narrative and systematic reviews on wine tourism activity, there is no previous bibliometric study that has analyzed the structure of scientific knowledge on wine tourism activity. However, despite the article's contributions, the study suffers from certain limitations. In this sense, only the Web of Science database was selected for the analysis, given that the aim was to prioritize quality over quantity. Furthermore, the study has the limitation inherent to bibliometric studies, given that the content of the works is not studied, but rather a quantitative analysis of the scientific production analyzed is carried out. On the other hand, the work stands out for its reproducibility, and can be followed up periodically if desired by the academic community interested in the subject of wine tourism. As a future line of research, the authors intend to carry out a bibliometric analysis of the scientific production on wine tourism and sustainability, in order to quantitatively analyze the scientific production on one of the main benefits of the academic literature on this activity.

## Author contribution statement

All authors listed have significantly contributed to the development and the writing of this article.

## Funding statement

This research did not receive any specific grant from funding agencies in the public, commercial, or not-for-profit sectors.

## Data availability statement

Data will be made available on request.

## Declaration of interest's statement

The authors declare no competing interests.

## Footnotes

1 Despite the WoS Core Collection has a control variable called “Research areas”, WoS Categories give a higher degree of division and, thus, potentially improve probabilities for deeper conclusions. However, due to the aim for standardization with other literature, WoS categories will be referred as research fields from now on.

2 Despite analysing both journals and conference proceedings together, only the term “journals” will be used from now on for easy reading. Consequently, “journals” includes both categories.

3 It must be kept in mind that one of the limitations of bibliometric analysis are the boundaries that have to be imposed. This topic is deeper addressed in the limitations section.
